# Abstracts and Gleanings

**Published:** 1887-08

**Authors:** 


### ABSTRACTS AND GLEANINGS.

**Intubation of the Larynx.**—Doubtless you are all familiar with the instruments of Dr. O'Dwyer and the mode of using them, at least in theory, if not in practice.

I will not occupy your time with a detailed description of the operation, but will call your attention to some particular points. It is necessary to have the child's arms secured, and the best way is to fold a sheet lengthwise about a foot wide and wrap it around the body, binding the arms securely to its side. Some one holds the child, and another assistant holds the child's head in the natural position, not thrown back. The mouth-gag is now introduced between the teeth, and it may be necessary to have this held, as some children throw it out even when carefully placed. You then pass the index finger of the left hand over the tongue, hook up the epiglottis, and feel the opening of the larynx. The handle of the applicator should be held near the child's sternum until the tube has reached the pharyngeal wall, when the handle is rapidly elevated and the tube directed downward and forward along the finger into the larynx. I believe it is better to pass the tube by the side of the finger instead of in front of it, as I formerly did, because in young children the epiglottis is flaccid, and the end of the tube may push it down and prevent the introduction of the tube. I think this has happened to me. But when you reach the end of the finger, you pass the tube directly *under* it, not over nor by the end of it. This is an important point.

When the tube is removed, the extractor is introduced under the finger in exactly the same manner. If the tube has been inserted into the larynx, respiration becomes easier. It is well to wait a minute or two before cutting the thread and withdrawing it, simply to be sure that the tube is in proper position.

There are several modifications of the O'Dwyer tubes, but I have no personal knowledge of any of them.

The relative value of tracheotomy and intubation as a means of saving life is shown by the following statistics:

Dr. A. Jacobi, of New York, who probably has had a larger experience than any other physician or surgeon in this country, reports that in over 400 tracheotomies that he has performed, only about a total of 12 per cent. have recovered.

Prof. Jacobi's statistics are more favorable than the average result, although I am aware that Drs. J. H. Ripley and Fred. Lange, of New York, report in 66 cases 33⅓ per cent. of recoveries.

Dr. Brush, the gentleman who first brought before the profession Dr. O'Dwyer's method, told me last February that he had intubated in eight cases, and all terminated fatally.

In a letter received from Dr. O'Dwyer, written April 8th, he says: "I have practiced intubation of the larynx since the beginning of my experiments in 1880, in 134 cases of croup, membranous or diptheritic, with 26 recoveries. During the first three years of this time, or while using very imperfect forms of tubes,

I had no recovery. I have operated in private practice now 69 times, with 17 recoveries." Thus it appears that Dr. O'Dwyer has had 19.4 per cent. of recoveries, including all of his cases, while in private practice, and since the use of his more perfect tubes, the recoveries have been 24.6 per cent. He also writes me that he has been called to operate upon five other cases of diphtheritic croup where he did not think it necessary, and the patients recovered without any operation.

Dr. Waxham reports (*Chicago Medical Journal and Examiner*, August, 1886,) that the record of recoveries in Chicago after tracheotomy has been 18.95 per cent., while that of intubation has been 27.71 per cent.

I have had four cases of intubation of the larynx, and although they all terminated fatally, there is no doubt in my mind but that one of them would have recovered had the child's mother obeyed the instructions given her, although the condition of the patient at the time of the introduction of the tube was very critical, and it was not expected that she could last long unless relief was afforded her. Upon the introduction of the tube, relief was almost immediate, and the child continued to improve for three days, when she was suddenly seized with a severe paroxysm of coughing and strangling, caused, as I believe, by drinking a glass of water, and suddenly expired.

In the other cases it was not expected, when the tubes were introduced, that recovery was possible, but in two of these cases the relief from the distressing dyspnœa was more than sufficient to justify the operation, and the parents of the children so expressed themselves.

In one case I was obliged to use too small a tube, the proper size being in the throat of another child at the time. The tube was coughed up and re-inserted three times without any apparent injury to the child.

It being true that the percentage of recoveries is greater from intubation than tracheotomy, and certainly there can be no doubt upon this point, it is much the preferable operation of the two. Anything which saves the life of a patient, or any procedure which saves more lives than another, is the proper one to adopt.

But besides the more favorable results, there are many other reasons which recommend intubation over tracheotomy.

Chief among these are that there is no objection upon the part of the parents, or, at least, but little ; and after it has been proposed and explained, the parents themselves usually ask for the introduction of the tube, if for any reason it be delayed.

In most cases, after the operation the relief is marvelous. Physicians of many years' experience say that they have never seen anything like it. The child usually expresses himself as greatly relieved, and in one case which I had, the patient said, soon after the introduction of the tube, "You are good doctors, to make me feel so much better."

If the child dies, there is no regret upon the part of the physician or friends that the operation was performed; usually only feelings of gratitude that the sufferings of the little patient have been so greatly relieved, even if his life has not been saved. Certainly, if the operation did no more than to relieve the terrible sufferings of the patient, it would, in my mind, be sufficient to fully commend it.

The tube is worn without much irritation, and expectoration occurs more readily than through the tube used in tracheotomy. The air that enters the lungs through the tube is warm and moist from its course through the upper air passages, and bronchitis or pneumonia is not as likely to occur as it is after tracheotomy.

In intubation, the operation is bloodless, and there is no open wound, which in itself may be a source of danger. It is more quickly performed. and with less danger than tracheotomy. If the patient recovers, convalescence is more rapid, the patient usually regaining the use of his voice in seven to ten days after the removal of the tube. Still another advantage is that the patient does not need the constant care of the surgeon or some skilled person, as in tracheotomy.

Although there are many reasons to recommend intubation, there are, as with everything else, several objections to it. One of these is the difficulty of introducing the tube, which in some cases, it must be confessed, is great ; yet, a sufficient amount of practice upon the cadaver, and a reasonable amount of dexterity, combined with coolness, there is usually no serious difficulty in the operation. If it is difficult, it is not a sufficient reason to prevent all being done to relieve the patient that is possible. Another objection is the inability of some patients to swallow liquids ; all can swallow solids and semi-solids without difficulty, and most patients can swallow liquids after a little practice without much trouble, if the nurse is careful to give it to them, at first, while lying on the side. In some cases the epiglottis does not seem to close over the tube, and then the child is unable to swallow liquids without coughing, indicating that some of the liquid has passed through the tube, and hence the dangers of lung complications are increased. Dr. O'Dwyer told me, if I understood him correctly, that he did not think there was much, if any, danger of any fluid passing through the tube in any case ; that he considers it safe to let a child drink a reasonable quantity of liquid if he wishes it. His experience is vastly greater than mine, yet I cannot but believe that the first case I had would have recovered if she had not been allowed to drink all she wished. This patient was doing very well, had greatly improved, and presented every indication of recovery. She could not, however, swallow liquids without coughing, unless she lay on her side, and took only a teaspoonful at a time.

Contrary to strict orders, the mother gave the patient a tumblerful of water, which she drank. She immediately had a violent fit of coughing and strangling, and in less than thirty minutes was dead.

Unfortunately, croup appears to occur more frequently in families where the parents are not remarkable for intelligence, or particular to follow closely the advice of the physician, and the care and attention which the patients receive is none of the best. After intubation the relief is usually so great that it is often very difficult to convince the friends that the child is still very ill; that you have only given it one more opportunity to get well—that it is yet suffering from a very grave disease, with the chances of recovery much against it. They are so pleased with the changed condition of the sufferer, who a few moments ago was on the verge of suffocation, that they cannot believe what you tell them, hence the patient does not receive the care and attention which he should. When we can have an intelligent nurse—one who will carefully attend to the administration or non-administration of food and drinks, and conscientiously carry out our instructions—and when the operation can be performed dexterously by the physician, I believe the recoveries from intubation will be much greater than at present reported, and the operation come into more frequent use.—*Dr. Ingraham in Buffalo Medical Jaurnal.*

**Vesical Calculi and the Different Methods Employed for Their Removal.**—Dr. A. Vandeveer, of Albany, New York, at American Surgical Association. read a paper on the above.

The essay was mainly made up of detailed histories of forty-one cases upon which he had operated. Referring to supra-pubic lithotomy, the author said he thought the operation would finally be limited to cases of large and, in some instances, of sacculated stone, and where, in male adults, there was chronic cystitis. On anatomical grounds the supra-pubic method is much simpler in the youth, because of the bladder being much higher in the pelvis at this time of life. He did not think that we would get from it as large percentage of recoveries. In the tables he had collected, embracing one hundred and forty-two cases in adults, the mortality was twenty-two per cent., and in one hundred and thirteen cases, in children under fifteen years, the mortality was a little over ten per cent.

He preferred litholapaxy where the stone is of moderate size, and said that, contrary to the teachings of a few years back, the operation can be safely done in young male children if proper instruments are used. He thought rapid dilitation of the urethra or supra-pubic lithotomy in girls would be sufficient. In adult women he would add in certain cases vaginal lithotomy.

Dr. W. T. Briggs, of Nashville, Tenn., opened the discussion of these several papers with the remark that he did not agree with one of the essayists, who predicted the time would come when the high operation and litholapaxy would be the only procedures adopted for the removal of stone. He fully agreed that in stones of great size, or in certain deformities of the pelvis and lower extremities, the supra-pubic method would at times be the best. He was unable to see, however, why in stones of medium size the perineal operation should be abandoned. He thought the best operation was that through the median line. The resistance which is usually met with at the neck of the bladder, he had found to be easily overcome by a lateral incision of three lines on each side of the prostate, when with gradual dilatation the opening can be enlarged sufficiently to allow the removal of any stone that should be removed through the perineum. He saw no reason why, after the bladder had been opened by this method and the stone found unusually large, it should not be crushed by the instruments in use for that purpose. He thought the operation done in the manner he had described, would give a smaller death-rate than litholapaxy. His first seventy-four cases all recovered. He then had two deaths in succession. In one, a pelvic abcess, and in the other, a very bad state of general health complicated the case. He then cut forty-six cases with but one death, this case dying three months after the operation, with general tuberculosis, the wound never having united.

Dr. D. Hayes Agnew, of Philadelphia, said we should not commit ourselves positively to any single operation. He regarded the median operation as safest of all procedures for the removal of calculi of ordinary size. He thought the only mischief likely to follow here was in extracting the stone, and this he felt could be avoided by nicking the neck of the bladder, which could then be distended to any desired amount. When the stone proves unduly large, the incision may be extended on either side of the prostate. He was very sure that drainage could be more readily effected in this than in the high operation. He agreed that in cases of large stone the high operation was best.

Dr. D. W. Yandell, of Louisville, Ky., remarked : I regret not having heard the early part of the discussion, for just at this time the subject is of special interest, and what is said of it here will have much weight when the evidence is finally summed up. I concur in large part with my distinguished friend on my left (Dr. Agnew). Although I was not present when Dr. Briggs made his remarks, I know pretty well what he said, for I know personally of his great success with the perineal operation. My own experience with three operations, lithotomy, lithotrity, and litholapaxy reaches 106 cases : 92 by the perineum, 8 by lithotrity, and 6 by litholapaxy. I have never done supra-pubic lithotomy, and have seen it done but twice. On neither of these occasions, though the operation was done on both by exceedingly clever surgeons, did it impress me as being superior to either the lateral or bilateral method. It did not seem so easy of execution ; it clearly required more time. And if injury to the virile powers of the patient is not considered, I am unable to see wherein it is safer. And to-day, with all the reports of successes both at home and abroad, and in spite of the very positive statements of the able men here who have advocated the procedure, I still see no sufficient reason why, in stones of ordinary size and character, I should abandon the methods I have hitherto followed. They have certainly seemed to me altogether sufficient. Seven per cent. only of my lithotomies ended fatally, and one of these cases died from erysipelas, and another from surgical kidney. It is true that most of the subjects were young persons, but still I had a due proportion among old men. In none of them was there a return of the stone. I have been less fortunate in this latter respect with both crushing operations, for I had two returns of stone in my eight lithotrities, and two ditto in the six Bigelows. I take it, however, that the four double operations were made necessary by the want of skill and experience on my part, rather than through any defect in the procedures themselves. I was no more successful than Dr. Vandeveer in the extraction of all the *debris*, and certainly, in the report he has just read, he has shown himself a capable man with both knife and lithotrite. To my mind the question is still *sub judice*, and must remain so until much more evidence has been accumulated. When calculi are too large to be taken out through the perineum, they should certainly be removed by opening the bladder at its fundus, and the procedure is clearly a safe one. When calculi are of suitable size and in suitable subjects they should, I think, certainly be crushed. When they are too hard to be crushed, the surgeon may choose between cutting above or below the pubes. And the choice is necessarily governed by many circumstances, none of which I need enumerate here. I will close with the remark that no one procedure for extracting stone from the bladder is applicable to all cases, and we should congratulate ourselves, therefore, that we possess so many means for accomplishing that purpose.

Dr. John B. Roberts, of Philadelphia, said: The high operation is certain to play a very important part in the future surgery of the bladder. It unquestionably permitted a much freer exploration of the bladder than when this organ was opened through the perineum, and he also thought that where the surgeon had no experience with either of the cutting operations, the supra-pubic method would prove the safest. He did not agree with Dr. Packard's suggestion in regard to treating retention of urine from stricture by supra-pubic cystotomy. He thought that long and persistent effort should be made by the surgeon to introduce a filiform bougie into the bladder, by which the urine in due time would drain away. Failing in this, simple aspiration above the pubes, which is both a safe and simple procedure, repeated at intervals for a few days, will almost always secure in a short time sufficient patency of the urethra for the introduction of instruments.

Dr. J. Michael, of Baltimore, agreed with the previous speakers as to the advisability of the supra-pubic operation for exploration, also in exceptional cases of foreign bodies in the bladder, and in certain cases of extreme prostatic enlargement. He thought, however, with Dr. Roberts, that in retention of urine due to ordinary stricture or prostatic disease, aspiration of the bladder was all that was needed.—*American Practioner and News.*

**Dr. Bergeon's Method of Treating Consumption by Means of Gaseous Enemata.**—Dr. Byron H. Daggett, of Buffalo, N. Y., read the following at the annual meeting of the Alumni Association, Medical Department, Niagara University, April 12th, 1887:

Being urged to carry out the treatment of pulmonary consumption as inaugurated by Prof. Bergeon, of France, and not having the apparatus for so doing, I attempted from a meagre description of the proposed method to extemporize an apparatus for fulfilling its indications. Messrs. Howell & Son kindly charged some of their so-called soda water bottles with carbonic dioxide. This carbonic gas bottle was connected by a rubber tube with a Wolff bottle containing nearly a quart of water saturated with hydrogen sulphide, and the Wolff bottle was again connected with a rubber bag having a stop-cock. Pressing upon the lever of the carbonic gas bottle, carrying with it the sulphuretted hydrogen into the bag, and when the bag is filled the stop-cock is turned and a rectal nozzle is introduced into the disconnected tube. The remedy is then ready for use, and is readily carried to the bedside of the patient. An objection made to this manner of generating and using the gas is that too much hydrogen sulphide may be used. My limited experience does not corroborate this view. I have found no ill effects from it, but, upon the other hand, my patients have soon noticed when the sulphurous water was depleted so as not to furnish the full complement of sulphurous gas, and would say, "It is not as strong as before and does not do as much good." Another objection has been made; that the bags would soon be destroyed. So far, I have not had any such trouble. This modification of the process certainly renders the treatment of patients much more convenient.

In 1882, Dr. Bergeon inaugurated the treatment of pulmonary consumption by gaseous enemata of carbon dioxide and hydrogen sulphide combined, and in July, 1886, reported wonderful results, so that this subject is attracting widespread attention.

Bernard demonstrated, in 1857, that toxic and medicinal agents introduced into the intestinal canal were taken up by the venous system, carried through the portal system, the liver, the hepatic veins and the lungs, and were eliminated by the liver and carried off by the bile, or, if volatile, were exhaled by the lungs. In these ways they were prevented from entering the arterial circulation.

He also demonstrated that hydrogen sulphide could be safely injected into the veins and rectum of a dog. Dr. Bergeon, experimenting upon animals, found that injecting preparations of chlorine, ammonia, ether, turpentine and bromine into the rectum caused violent inflammation.

He introduced a mixture of carbon dioxide and hydrogen sulphide, and learned that this combination was well borne, if free from atmosperic air. Reasoning from these premises and knowing that sulphur is an antiseptic as well as a microcide, he instituted the treatment of pulmonary maladies by gaseous enemata. He reported over two hundred cases treated in this way, and some of them he considers cured.

Dr. Cohen, of Philadelphia, says: "All reported cases show rapid amelioration of all suppurative phenomena; marked diminution of cough, expectoration, dyspœna and night sweats being noted within two or three days."

In some of his own cases, there was a similar prompt improvement, but not in all, although they have all done well with one exception; some were very pronounced cases, and there was no hope in the more familiar methods of treatment. Dr. Bruen, after several weeks' treatment of cases in the various stages of the disease, summarizes as follows :

"In nearly all cases, lasting effects have been secured in the reduction of temperature, suspension of night sweats, lessened cough and expectoration, and in some *all physical signs of bronchial catarrh are abolished.*

"The amount of gas introduced has been from three quarts to a gallon at each injection, taking from fifteen to thirty minutes.

"Injections were given twice daily in most cases.

"No injurious effects were observed.

"Effects were negative in only one case.

"The ultimate value of the treatment can only be established by time.

"The mode of action would seem to be antiseptic, and by reducing suppuration, consequent relief of serious symptoms, permitting the patient to gain by food, exercise and general treatment.

"Thus far the gas appears to be a valuable therapeutic agent, rather than a curative plan of treatment."

In this, Bruen appears to differ with Bergeon who, after four years' experience, reports specifically, and in detail, individual cases who are well and who present no symptoms of the disease, and whom the doctor regards as cured.

My experience in cases in the past ten days coincides with these reports in this : there is marked amelioration of all the symptoms. In the first case, Mr. W., a severe diarrhœa ceased after beginning this treatment. Pulse rate was reduced from 140 to 90 in three days, respirations from 40 to 24. The sputa became thinner and colored yellowish. There was marked relief to the pain and constringency of the chest. One case reports a cold sweat, followed by a severe chill, the third night after beginning the treatment. With this exception, there has been no night sweats since the second night. My patients lose the hectic flush and look pale. The chill referred to I attributed to the treatment, in part at least, as I had pushed the remedy beyond the regulation amount. In administering the gas, I have noticed that there will be a complete stasis for three or four minutes, and suddenly the bag will rapidly subside with a gurgling noise ; this brings on colic, and the gas should be turned off until the distension of the abdomen subsides, which will be three or four minutes more. I have administered from three to six quarts once and twice per day, as I found it convenient. My patients send for the bags after three or four treatments, and administer it themselves, at their own pleasure. My experience is too brief and limited to warrant me in formulating deductions of any results except temporary relief.

This method of Bergeon opens a vast field for investigation, and another avenue for reaching and, we trust, successfully combatting, lung as well as other visceral maladies.—*Buffalo Medical and Surgical Journal.*

**The Cure of Boils by Injection.**—Dr. Bidder described, at a recent meeting of the Berlin Medical Society, a new method of treating furuncles by parenchymatous injections of carbolic acid. If the boil is a small one, he gives one injection of a few drops of a solution of carbolic acid (2 per cent.); if it is of medium size, two injections are given, the half of the whole of a Pravaz syringeful of the solution being used on each occasion. In the case of large furuncles, for example, half the size of a man's hand, Dr. Bidder injects, at four different spots, the contents of four Pravaz syringes half or wholly filled with a solution of 2 per cent. of carbolic acid. These injections are given only once. This treatment is strikingly successful. There is some smarting at the seat of injection at first, but the pain soon disappears, and the next day there is a marked improvement in the patient's condition. The inflammatory swelling subsides very quickly, and in eight or ten days even the largest furuncle is dispersed. By this plan no unsightly scars are left, a circumstance which in many cases is of considerable importance. The success of the treatment is probably to be accounted for by the fact that either the microbes which cause the disease are killed or the medium in which they flourish is destroyed.—*Ex.*

**Antifebrin.**—Of all the modern antipyretics, antifebrin appears to take the lead, and will without doubt replace quinine, thallin, kairin, and even antipyrin. We make the following excerpts from Professor Wm. Osler's excellent article published in the *Therapeutic Gazette:* "Antifebrin is known chemically as acetamide or acetanilide. It is a neutral body, and in this respect it differs from all other antipyretics, which are either phenols, like salicylic acid and resorcin, or bases of the chinoline series, as thallin, antipyrin, and quinine. It is a white crystalline powder, insoluble in cold water, but readily dissolving in hot water or alcoholic solutions. The taste is not unpleasant. The dose is from 8 to 12 grains. In large amounts it is not poisonous, though it is not advisable to exceed 30 grains in the day. Usually 8 grains will be found an effective dose. It is conveniently given in spirits and water, or in whisky, or, for children, in warm sweetened water.

"For brevity, the effects of the drug may be noted under the following heads:

1. *Reduction of Temperature.*—"This is the marked and characteristic action, beginning usually within an hour. In eighteen administrations the fall was over 2° in this time; in three instances a fall of 3°; on two occasions a fall of 4°. In thirteen instances the temperature was reduced 4° in two hours; in sixteen administrations 3°; and on four occasions 5°.

"The greatest drop within this time was in case XXIV, in which the fall was 6 2-5°. The greatest reduction was in the following: Case I, 8° in five hours; case X, 6 3-5° in five and one-half hours; case XVIII, 7 3-5° in two and one-half hours; case XX, 7° in seven hours; case XIX, 7 3-5° in ten hours.

"In the erysipelas cases the action was in each instance most decided.

"In phthisis, with high fever, the drug was usually given in a single powder of 8 grains, when the temperature was above 103°, but in three cases the plan was tried of giving 4 grains four or five times a day. This did not seem very successful, and the patients did not feel so comfortable as with the single dose.

"In a remarkable case of quartan ague, antifebrin in 8-grain doses given before or during the paroxysm seemed to be without effect. One curious circumstance, however, is worth mentioning. The lad had always with the fever the most intense general urticaria, which the antifebrin seemed to prevent, much to the patient's comfort.

2. *Action on the Circulatory System.*—"Usually with the reduction of the fever the pulse would fall, and a drop of 20 or 30 in two or three hours was frequently noted. Thus in case II, with a pulse-rate of 112 per minute, and the temperature at 105°, the pulse fell to 84 in four hours. In another case the pulse fell from 130 to 90 in four hours. A marked increase in the pulse-tension was observed in several cases. Even with a rapid fall of from 5° to 7° in two or three hours, there was no evidence of heart weakness. Slight cyanosis, which is mentioned by one or two German writers, did not occur in any instance.

3. *Sweating.*—"As with thallin and antipyrin, the action of antifebrin is almost invariably accompanied with profuse perspiration, which is often the first effect of the drug. Repeatedly I have seen the forehead beaded with sweat half an hour after the giving of 8 grains. This is sometimes a most unpleasant feature in the employment of the drug, and is the only one of which the patients have complained. In several instances the drug was combined with atropine, but without much effect. It does not seem to increase the night-sweats in case of phthisis; indeed, under its use one patient, who sweated much with the afternoon dose, had drier and, in consequence, more comfortable nights. In the severe typhoid case already referred to, I stopped its use, as the sweating seemed to weaken the patient.

4. *On the Urine.*—"The only change noticed was a marked increase in the amount in some of the cases. This is probably a direct result of the increased arterial tension.

5. "The effect on the general condition seemed usually beneficial. A quiet sleep often followed an hour or so after its administration. The phthisical patients expressed themselves more positively than others in this matter.

"There were none of the disagreeable effects which we sometimes see follow the use of antipyrin and thallin. There was no instance of vomiting; and, with the exception of case IV, there was no shivering or chilliness, such as is common after antipyrin.

"These limited observations confirm those of Cahn and Hepp, and others, and I think that we have in antifebrine a prompt and powerful antifebrile agent, easy to take, and free from unpleasant effects It has the advantage also of cheapness. Merck's article, which I have used, is only 60 cents an ounce, wholesale.—*National Druggist.*

**Water for Babies.**—Dr. Touissaint, in an article in the *Union Medicale de Canada*, calls attention to the fact that milk does not satisfy the thirst of babies. It appeases hunger, but it frequently intensifies thirst, and the author maintains that it is this very thirst that causes healthy children, raised altogether at the breast, to cry so frequently and so violently. We have seen peevish, fretful infants, upon whom all the arts of the nurse and mother had in vain been tried without eliciting a smile, suddenly brighten up at the sight of water, reach eagerly for it, and on obtaining a drink, go off to sleep calmly and contentedly. We quite agree with Dr. Touissaint when he declares that many cases of infantile indigestion would be benefitted or cured by giving the little patients a regular supply of water.—*St. Louis Medical and Surgical Journal.*

[Right; you hit the nail on the head, sure.—ED. RECORD.]

**The Cure of Epilepsy.**—In the January number of the *Alienist and Neurologist*, the reporter maintains the curability of epilepsy on the following conditions:

No epileptic can be cured who persists in the use of tobacco, alcohol or other depressing narcotic, or vicious habit, or who does not give up coffee and tea and learn to use milk and a minimum of animal food, or to use tea and coffee but very sparingly in the forepart of the day only.

He must have secured to him the habit of sleeping abundantly and quietly, and avoid all sources of passionate outbursts.

His bowels should be kept uniformly regular on a laxative pill, containing aloin, colocynth and rhubarb extract, or blue mass, as indicated at night, with an eighth of a grain of extract of belladonna and a fourth of a grain of extract of conium.

The indications for the successful management of epilepsia are to put the general and whole glandular system in physiological working order, remove all sources of eccentric nerve irritation, and duly tranquilize and reconstruct the irritable cerebral centres, keeping up the treatment till all tendency to psychical or motor explosion in the cerebral centres disappears, if it takes a lifetime to do it.

The cephalic galvanization should be employed at least three times a week, with a descending and labile current, until improvement in the cerebral nerve-tone is accomplished and confirmed. Ordinarily a four to six cell current from a simple McIntosh battery will suffice.

He considers skillfully employed cephalic galvanization a most essential remedy for the permanent cure of epilepsia, notwithstanding it has been disparaged by those who ought better to understand its use and powers before condemning it. The same is true in his experience with its use in chorea, and all central neuropathia in which morbid irritability and a tendency to "explosive" cell action—motor or psychical, as in active mania, the paroxysms of hysteria, tetanus and hydrophobia. He sums up the matter as follows:

When the cure of epilepsy is possible by medical means, and it is often possible to cure this disease if we treat it properly, it is necessary in every case to adopt a plan, which consists of—

1. A judicious combination of cerebral reconstructives, conservators and tranquilizers of nerve-force and regulators of explosive action, epilepsy being a discharging lesion—the brain of an epileptic expending its accumulated psychical and motor force during a paroxysm, much as a spring and pendulum clock expands its power by suddenly running down all at once, whenever the pendulum or regulating ("inhibitory force") is removed.

2. The control of the cerebral vasomotor system through persistent galvanizations (never faradizations). The galvanizations of epilepsy are with us, in some cases, an everyday affair (so to speak) for several months—short seances, but long continued treatments.

3. The regulation of all the patient's habits, moral, social, psychical and physical ; as much so as if he were a patient in the insane asylum.

To do this, the patient must be studied and treated daily for a while, and must be as well understood by the physician as a case of pneumonia or typhoid.

We cure epilepsy in this way—not always, but quite often—often enough to satisfy us well for our pains.—*Weekly Medical Review.*

**Treatment of Certain forms of Vomiting.**—There are few disorders which cause more discomfort and distress than those accompanied with incessant attacks of vomiting; there are few disorders which try more the patience and the skill of the practitioner. Dr. F P. Atkison gives us in the *Practitioner* (for Nov., 1886,) a number of points which may prove useful in relieving certain forms of obstinate vomiting.

In case of simple bilious vomiting he states that a mixture containing 15 minims of solution of potassium and 4 of laudanum administered every four hours acts like a charm, and he asserts that in no uncomplicated cases will there be any vomiting after two or three doses. For the vomiting of pregnancy he suggested a little milk and tea, with a small piece of bread and butter or biscuit, immediately before rising in the morning, and a biscuit or two at various intervals throughout the day, whenever there is a feeling of emptiness. In vomiting from ulceration of the stomach as much rest as possible. This may be accomplished by giving very small quantities of peptonized milk or koumiss at short intervals; thus a teaspoonful of the above may be mixed with cold water and given every four hours. Dr. Atkison further recommends that the body should be oiled night and morning to help nutrition, and covered with warm clothing to prevent cold. Later on, when the pain has almost subsided, various simple foods may be allowed. When the vomiting is very urgent, of course the stomach should be given entire rest, and peptonized meat enemata should be administered.

In the vomiting which occurs in infants brought up by hand, the most frequent cause is found in their inability to digest the casein of the milk. In such cases it is advisable to use one of the many peptonizing powders now on the market, or the following may be given: Two tablespoonsful of whey, 2 tablespoonsful of water, and one of cream; if there be some diarrhœa, a little meat juice may be given three or four times daily, while the body should be oiled night and morning.—*Technics.*

**Medicinal Eruptions.**—The list of drugs capable, when taken internally, of exhibiting an impression upon the skin, has grown in numerical proportions within a few years past. Arsenic and belladonna and iodide of potassium no longer share the honor of being the chief eruptive disturbers of the system. Chloral and opium, digitalis, quinine, salicylic acid, carbolic acid, copaiba, mercury, bromide of potassium, turpentine, and a number of other agents, of dissimilar therapeutic efficacy, claim analogous properties. How much longer the list will become, under the future investigations of the dermatologists, time will alone determine. It may well be questioned whether many of the cutaneous diseases with which the general practitioner and even the specialist have been embarrassed, have not frequently been the effects of remedies already administered, rather than original morbid affections ascribable to other causes. How many of the diseases of the skin under daily observation, even in these advanced times, may not be due to the ingestion of iodide of potassium, digitalis, or some other remedy, struggling to make itself publicly apparent on the surface of the body! Would it not be as well for the menders or amenders of the nomenclature of cutaneous diseases to inquire, before coining new names for strange affections, whether the eruption may not be due to the influence of some such potent remedy? We remember, some time since, noting a suggestion made by one of our contemporaries as to the evil consequences to be apprehended if two unprincipled members of the profession should act in collusion, one of them dispensing in his practice medicines which, if perseveringly administered, would certainly produce decided cataneous manifestations; the other a specialist in skin diseases, who would follow his co-conspirator after an interval of a few brief weeks, the time being graduated to the exigency of the case, and administer the remedy exactly suited to the erythema or other exanthem with which the patient might be afflicted. Seriously, however, it seems to be well established that a number of eruptions of varied character may appear, consequent upon the employment of quinine. The practitioner cannot be too careful in forming a proper diagnosis in these cases, so that he may avoid perseverance in a medicinal agent which would only tend to aggravate the existing morbid condition and the special determination to the skin.—*College and Clinical Record.*

**The Last Treatment of Phthisis.**—The very latest treatment of phthisis is by large doses of tannin. Scarcely had we begun to grasp the details of the medicated gaseous enemata process, as practiced in the French hospitals, before Drs. Arthaud and Raymond announce something simpler and better. We learn from the Paris letter to the *British Medical Journal*, that these gentlemen began experimenting on rabbits, in order to find some substance that would render them non-susceptible to inoculations of tuberculous matter. They obtained some very remarkable results from tannin.

"Six rabbits were treated for a month with doses of tannin, varying from seven and one-half to fifteen grains; after two successive inoculations, one with lung-tissue from a patient who had died of acute tuberculosis, the other with miliary tubercle from a hospital patient—no trace of infection was observed, while the three rabbitts, to which tannin had not been given, succumbed in consequence of inoculations with the same material. These experiments suggested a mode of treatment which has been adopted with excellent results in over fifty cases. Tannin was given in doses of 30 to 60 grains a day, and the improvement was visible at the end of a fortnight, the patients had increased in weight, and no relapse occurred. In cases of acute tuberculosis, both in children and adults, it sometimes happens that the symptoms appears less favorable; but at the end of a week or a fortnight, the patient's condition improves, even when fatal results have been feared. From these experiments the following conclusions may be drawn:

1. The tannin is preferable to sulphide of carbon or iodoform in the treatment of tuberculosis.

2. That animals submitted to this treatment for a month offer great resistance to the action of tubercular virus."—*Southwestern Medical Gazette.*

**Treament of Sciatica.**—Dr. J. T. Metcalf says: "Some months ago I got my druggist to make tablet-triturates, each of which contain three drops of the following mixture:

℞. Tincture of aconite root..................
Tincture of seeds of Colchicum...........
Tincture of belladonna..... ............
Tincture actea racemosa....... ...Equal parts by volume.

Of these I give one every four, six or eight hours, according to the necessity of the case. It takes not long to impress the system, especially with the aconite, nor does it as a rule, require more than three days to make the patient so firmly believe in its efficacy that he has no need of persuasion to insure its continuance until complete relief.

In neuralgia of the axillary and brachial nerves, it has proved quite as efficacious as in that of the great crural branch."—*Boston Medical and Surgical Journal.*

**Treatment of Sciatica.**—Dr. Metcall, of New York, says that no prescription for sciatica has ever equaled in efficiency the following: ℞. Tinct. aconit. rad., tinct. colchic. sem., tinc. belladonna, aa ʒ j. M. Sig. Dose, 6 drops every six hours. He also uses triturate tablets, each containing thre drops of the following: Tincture of aconite root, tincture of aconite root, tincture of seeds of colchicum, tincture of belladonna, tincture of actea racemosa—equal parts by volume. Dose, one every four to eight hours.—*Journal of American Medical Association.*

